# New lager yeast strains generated by interspecific hybridization

**DOI:** 10.1007/s10295-015-1597-6

**Published:** 2015-02-15

**Authors:** Kristoffer Krogerus, Frederico Magalhães, Virve Vidgren, Brian Gibson

**Affiliations:** 1 VTT Technical Research Centre of Finland, Tietotie 2, P.O. Box 1000, 02044 Espoo, Finland; 2 Department of Biotechnology and Chemical Technology, School of Chemical Technology, Aalto University, Kemistintie 1, Aalto, P.O. Box 16100, 00076 Espoo, Finland

**Keywords:** Lager yeast, *S. eubayanus*, Brewing, Hybrid, Mating, Heterosis

## Abstract

The interspecific hybrid *Saccharomyces pastorianus* is the most commonly used yeast in brewery fermentations worldwide. Here, we generated de novo lager yeast hybrids by mating a domesticated and strongly flocculent *Saccharomyces cerevisiae* ale strain with the *Saccharomyces eubayanus* type strain. The hybrids were characterized with respect to the parent strains in a wort fermentation performed at temperatures typical for lager brewing (12 °C). The resulting beers were analysed for sugar and aroma compounds, while the yeasts were tested for their flocculation ability and α-glucoside transport capability. These hybrids inherited beneficial properties from both parent strains (cryotolerance, maltotriose utilization and strong flocculation) and showed apparent hybrid vigour, fermenting faster and producing beer with higher alcohol content (5.6 vs 4.5 % ABV) than the parents. Results suggest that interspecific hybridization is suitable for production of novel non-GM lager yeast strains with unique properties and will help in elucidating the evolutionary history of industrial lager yeast.

**Electronic supplementary material:**

The online version of this article (doi:10.1007/s10295-015-1597-6) contains supplementary material, which is available to authorized users.

## Introduction

Pale lager is the most popular beer style worldwide. All beers of this type are produced through the fermentation of malt wort at low temperature with the interspecific hybrid yeast species *Saccharomyces pastorianus* [14]. The formation of interspecific hybrids is common among the closely related species of the *Saccharomyces* genus, and the lager yeast *S. pastorianus* is a natural hybrid between *S. cerevisiae* and the newly discovered *S. eubayanus* [21]. Hybrid species are common in anthropogenic environments and are not limited to yeast, as hybrid species often exhibit qualities superior to both parent strains [22, 33]. Lager beers have gained popularity due to their ‘clean’ flavour profile, i.e. lack of ester-derived fruity/floral aroma, resulting mainly from fermentation at low temperatures. Ale strains (*S. cerevisiae*) have over time been selected for beneficial brewing properties, such as flocculation, sugar utilization, and flavour profile, but require warmer temperatures (>15 °C) for optimal fermentation [40]. In contrast, *S. eubayanus* thrives at the low temperatures responsible for the typical aroma profile of lager beer, but lacks the ability to utilize maltotriose (the most abundant sugar in wort after maltose) and exhibits poor flocculation [13].

The origins of lager yeast are not known, but it may be possible that the original hybridization event was due to *S. eubayanus* contaminating a traditional ale fermentation with *S. cerevisiae* [14]. The *S. pastorianus* group consists of at least two distinct lineages (Saaz and Frohberg), which may have arisen independently [11, 23] or through a common hybridization event [42]. While these two groups share many common features, they differ functionally in a number of respects, such as maltotriose utilization and cold tolerance [13, 34]. These functional differences seem to be reflected in the genomic difference between the groups (Saaz strains have retained proportionally more DNA derived from the *S. eubayanus* parent, while Frohberg strains have retained proportionally more DNA from the *S. cerevisiae* parent). Because lager yeasts are restricted to only these two genetically distinct groups, the genetic diversity among them is poor. However, through the selection of individual *S. eubayanus* and *S. cerevisiae* strains with specific desirable characteristics, and subsequent hybridization of these strains, it may be possible to produce novel, tailor-made yeast strains immediately available for industrial use.

The use of interspecific hybridization for generating ale and wine yeast strains with improved aroma production has been described [1, 12, 38, 39]. We here describe, for the first time, the use of mass mating to generate de novo lager yeast hybrids from a domesticated strongly flocculent *S. cerevisiae* ale strain and the *S. eubayanus* type strain. The hybrids were characterized with respect to the parent strains in a wort fermentation performed at temperatures typical for lager yeast, and the resulting beer was analysed for sugar content and the yeast was tested for its flocculation ability. The aim of this study was to demonstrate the generation of novel lager yeast strains with physiological properties inherited from both parental strains. This technique has the potential to greatly increase the diversity of yeast strains available for lager brewing [14]. The yeast hybrids generated with this technique can also be seen as non-GM, and their use is thus not prevented by legislation or public opinion [6].

## Materials and methods

### Yeast strains

The two parental strains were *S. cerevisiae* VTT-A81062 (VTT Culture Collection, Finland), a brewer’s yeast strain originally sourced from an ale beer from the United Kingdom, and the *S. eubayanus* type strain VTT-C12902 (VTT Culture Collection, Finland; deposited as CBS12357 at CBS-KNAW Fungal Biodiversity Centre). The four hybrid strains (A81062 × C12902) that were chosen for further characterization were named H1–H4. Natural auxotrophic mutants (*lys*- and *ura*-) of the parental strains were selected on α-aminoadipic and 5-fluoroorotic acid agar plates, respectively [4, 50]. Auxotrophy was confirmed by the inability to grow on minimal selection agar medium (0.67 % Yeast Nitrogen Base without amino acids, 3 % glycerol, 3 % ethanol and 2 % agar).

### Sporulation

For the generation of ascospores, the auxotrophic mutants of the parental strains were first grown overnight in YPM medium (1 % yeast extract, 2 % peptone, 4 % maltose) at 20 °C. The yeast was then inoculated into pre-sporulation medium (0.8 % yeast extract, 0.3 % peptone, 10 % glucose) at an OD600 of 0.3 and allowed to grow for 20 h at 20 °C. The yeast was then washed with 1 % potassium acetate and a thick suspension was plated onto sporulation agar (1 % potassium acetate, 10 mg/L lysine and uracil, 2 % agar). The yeast was allowed to sporulate for 7 days at 25 °C. Sporulation efficiency was calculated by counting the frequency of ascospores stained with malachite green [27]. Spore viability was calculated by dissecting ascospores treated with Zymolyase 100T (US Biological, USA) on YPD agar with a micromanipulator [43].

### Generation of interspecific hybrids

Interspecific hybrids between a *ura*- isolate of *S. cerevisiae* A81062 and a *lys-* isolate of *S. eubayanus* C12902 were produced by first generating ascospores of the auxotrophic mutants as above. Ascospores were scraped off the agar into 1 ml sterile reverse-osmosis purified H_2_O in 2 ml Eppendorf tubes. Tubes were centrifuged at 5,000×*g* for 5 min and the supernatant was removed. Ascus walls were digested by the addition of 50 μl 1 mg/ml Zymolyase 100T and incubation at 30 °C for 20 min. 200 μl of sterile H_2_O was then added, and cells were resuspended by vortexing. 100 μl of the resulting suspensions from both parental strains, with complementary auxotrophic markers, were transferred together to 1 ml YPM medium in a sterile 2 ml Eppendorf tube. Tubes were vortexed and incubated statically at 25 °C for 7 days. After incubation, the tubes were centrifuged at 5,000×*g* for 5 min and the supernatant was removed. 500 μl of starvation medium (0.1 % yeast extract and 0.1 % glucose) was added, and tubes were incubated for at least 2 h at room temperature. Tubes were then vortexed, after which the approximate cell concentration of the resulting suspension was measured with a NucleoCounter YC-100 (ChemoMetec, Denmark) and 100 μl aliquots were spread onto minimal selection agar (without uracil or lysine). Plates were incubated at 25 °C, and prototrophic colonies (i.e. potential hybrids) appeared after 3–7 days. Colonies were counted and purified by replating on minimal selection agar.

### Confirmation of hybrid status by PCR and RFLP

The hybrid status of isolates was confirmed by amplification of rDNA-PCR (ITS1, 5.8S and ITS2) using the primers ITS1 (5′-TCCGTAGGTGAACCTGCGG-3′) and ITS4 (5′-TCCTCCGCTTATTGATATGC-3′) and digestion of amplicons using the *Hae*III restriction enzyme (New England BioLabs, USA) as described previously [31]. Identification was based on the number of restriction fragments generated by enzyme digestion. *S. eubayanus* yielded a 3-band pattern (490, 225, 140 bp), *S. cerevisiae* a 4-band pattern (320, 225, 180, 140 bp), while successful hybrids yielded a pattern with all 5 bands (490, 320, 225, 180 and 140 bp).

Amplification of the *S. eubayanus*-specific *FSY1* gene (amplicon size 228 bp) and the *S. cerevisiae*-specific *MEX67* gene (amplicon size 150 bp) was also performed on the DNA extracted from the hybrid strains using the primers SeubF3 (5′-GTCCCTGTACCAATTTAATATTGCGC-3′), SeubR2 (5′-TTTCACATCTCTTAGTCTTTTCCAGACG-3′), ScerF2 (5′-GCGCTTTACATTCAGATCCCGAG-3′), and ScerR2 (5′-TAAGTTGGTTGTCAGCAAGATTG-3′) as described by Muir et al. [28] and Pengelly and Wheals [30]. Hybrids were i€dentified by the presence of both genes.

### Confirmation of hybrid status by PFGE

Yeast strains were propagated in YPM at 20 °C to an OD600 >1 and then harvested by centrifugation (3,000×*g*, 5 min, 4 °C). Supernatants were decanted, and cells were resuspended in 10 ml of 4 °C 50 mM EDTA (pH 8). Cell concentrations were determined with a Nucleocounter^®^ YC-100™ (ChemoMetec) and 1.2 × 10^8^ cells were placed in each sample plug. Sample plugs were prepared with the CHEF Genomic DNA Plug Kit for Yeast (Bio-Rad) according to the manufacturer’s instructions.

Sample plugs were loaded into the wells of a 1.0 % pulse field certified agarose (Bio-Rad) gel. PFGE was performed at 14 °C in 0.5× TBE buffer [89 mMTris, 89 mM boric acid, 2 mM EDTA (pH 8)]. A CHEF Mapper XA pulsed field electrophoresis system (Bio-Rad) was used with the following settings: 6 V/cm in a 120° angle, pulse length increasing linearly from 26 to 228 s, and total running time of 38 h. A commercial chromosome marker preparation from *S. cerevisiae* strain YNN295 (Bio-Rad) was used for molecular mass calibration. After electrophoresis, the gels were stained with ethidium bromide and scanned with Gel Doc XR+ imaging system (Bio-Rad).

### DNA content by flow cytometry

Flow cytometry was performed on the yeast strains essentially as described by Haase and Reed [16]. Cells were grown overnight in YPD medium (1 % yeast extract, 2 % peptone, 2 % glucose), and approximately 1 × 10^7^ cells were washed with 1 mL of 50 mM citrate buffer. Cells were then fixed with cold 70 % ethanol and incubated at room temperature for 1 h. Cells were then washed with 50 mM citrate buffer, resuspended in 50 mM citrate buffer containing 0.25 mg mL^−1^ RNAse A and incubated overnight at 37 °C. 1 mg mL^−1^ of Proteinase K was then added, and cells were incubated for 1 h at 50 °C. Cells were then stained with SYTOX Green (2 μM; Life Technologies, USA), and their DNA content was determined using a FACSAria cytometer (Becton–Dickinson). DNA contents were estimated by comparing fluorescence intensities with those of *S. cerevisiae* haploid (CEN.PK113-1A) and diploid (CEN.PK) reference strains. Measurements were performed on duplicate independent yeast cultures, and 100,000 events were collected per sample during flow cytometry.

### Characterization of hybrid strains

Four randomly selected hybrids (H1–H4) and the parental strains were chosen for further characterization in a small-scale wort fermentation performed at 12 °C. Yeast was propagated essentially as described previously [19], with the use of a ‘Generation 0’ fermentation prior to the actual experimental fermentations. The experimental fermentations were carried out in duplicate, in 2-L cylindroconical stainless steel fermenting vessels, containing 1.5 L of wort medium. The wort was produced at the VTT Pilot Brewery from barley malt and wheat malt, and contained an extract content of 12.0 ° Plato (59 g maltose, 19 g maltotriose, 16 g glucose, and 4.6 g fructose per litre) and free amino nitrogen (FAN) content of 269 mg L^−1^. Yeast was inoculated at a rate of 4 g fresh yeast per litre of wort (corresponding to 16  ×  10^6^ viable cells mL^−1^). The wort was oxygenated to 18 mg L^−1^ prior to pitching. The fermentations were carried out at 12 °C for 11 days, or until no change in residual extract was observed for 24 h. Wort samples were drawn regularly from the fermentation vessels with a syringe, and placed directly on ice, after which the yeast was separated from the fermenting wort by centrifugation (9,000×*g*, 10 min, 1 °C).

Flocculation of the yeast strains was evaluated using a modified Helm’s assay essentially as described by D’Hautcourt and Smart [9]. Cultures recovered from fermentation were washed twice with 0.5 M EDTA (pH 7) to break the cell aggregates and then diluted to an OD600 of 0.4. Flocculation was assayed by first washing yeast pellets with 4 mM CaCl_2_ solution and resuspending them in 1 ml of flocculation solution containing 4 mM CaCl_2_, 6.8 g/L sodium acetate, 4.05 g/L acetic acid, and 4 % (v/v) ethanol (pH 4.5). Yeast cells in control tubes were resuspended in 0.5 M EDTA (pH 7). After a sedimentation period of 10 min, samples (200 μL) were taken from just below the meniscus and dispersed in 10 mM EDTA (800 μL). The absorbance at 600 nm was measured, and percentage of flocculation was determined from the difference in absorbance between control and flocculation tubes.

Maltose and maltotriose uptake by the yeast strains was assayed by first growing them in YPM medium at 20 °C. Yeasts were usually harvested at an OD600 nm between 4 and 8 (i.e. at 2 ± 1 mg dry yeast mL^−1^) by centrifugation, washed with ice–cold water and then with ice–cold 0.1 M tartrate-Tris (pH 4.2) and finally resuspended in the same buffer at a concentration of 200 mg of fresh yeast mL^−1^. Zero-trans rates of [U-^14^C]-maltose and [U-^14^C]-maltotriose uptake at 20 °C were determined with 5 mM substrate in 0.1 M tartrate-Tris (pH 4.2) as described earlier [24], with reaction time of 1 min. [U-^14^C]-maltose (ARC 488) and [U-^14^C]-maltotriose (ARC 627) were from American Radiolabeled Chemicals Inc. (St. Louis, MO, USA). [U-^14^C]-maltotriose was repurified before use as described by Dietvorst et al. [10].

### Wort and beer analysis

The specific gravity, alcohol level and pH of samples were determined from the centrifuged and degassed fermentation samples using an Anton Paar density meter DMA 5000 M (Anton Paar GmbH, Austria) with Alcolyzer Beer ME and pH ME modules (Anton Paar GmbH, Austria).

The yeast dry mass content of the samples (i.e. yeast in suspension) was determined by washing the yeast pellets gained from centrifugation twice with 25 mL deionized H_2_O and then suspending the washed yeast in a total of 6 mL deionized H_2_O. The suspension was then transferred to a pre-weighed porcelain crucible and was dried overnight at 105 °C and allowed to cool in a desiccator, before the change of mass was measured.

Concentrations of fermentable sugars (glucose, fructose, maltose and maltotriose) were measured by HPLC using a waters 2695 separation module and waters system interphase module liquid chromatograph coupled with a waters 2414 differential refractometer (Waters Co., Milford, MA, USA). An Aminex HPX-87H organic acid analysis column (300 × 7.8 mm, Bio-Rad) was equilibrated with 5 mM H_2_SO_4_ (Titrisol, Merck, Germany) in water at 55 °C and samples were eluted with 5 mM H_2_SO_4_ in water at a 0.3 ml/min flow rate.

Yeast-derived flavour compounds were determined by headspace gas chromatography with flame ionization detector (HS-GC-FID) analysis. 4 mL of samples was filtered (0.45 µm), incubated at 60 °C for 30 min and then 1 mL of gas phase was injected (split mode; 225 °C; split flow of 30 mL min^−1^) into a gas chromatograph equipped with a FID detector and headspace autosampler (Agilent 7890 Series; Palo Alto, CA, USA). Analytes were separated on a HP-5 capillary column (50 m × 320 µm × 1.05 µm column, Agilent, USA). The carrier gas was helium (constant flow of 1.4 mL min^−1^). The temperature program used 50 °C for 3 min, 10 °C min^−1^ to 100 °C, 5 °C min^–1^ to 140 °C, 15 °C min^−1^ to 260 °C and then isothermal for 1 min. Compounds were identified by comparison with authentic standards and were quantified using standard curves. 1-Butanol was used as internal standard.

## Results and discussion

### Generation of interspecific hybrids

Auxotrophic mutants of the parental strains were successfully generated and the mutant strains, A81062 *ura*- and C12902 *lys*-, produced ascospores on the solid sporulation medium. However, quite low sporulation efficiencies were observed for the A81062 *ura*- strain compared to the C12902 *lys*- strain (11 and 78 %, respectively). These ascospores did contain viable spores though (12 and 71 %, respectively) and were used to generate the interspecific hybrids. After mating and spreading on selection plates, the first colonies started to emerge after 3 days. After a week, a total of 38 colonies (corresponding to a hybridization frequency of 2.6 × 10^−6^) were obtained.

### Confirmation of hybrid status

**Fig. 1:**
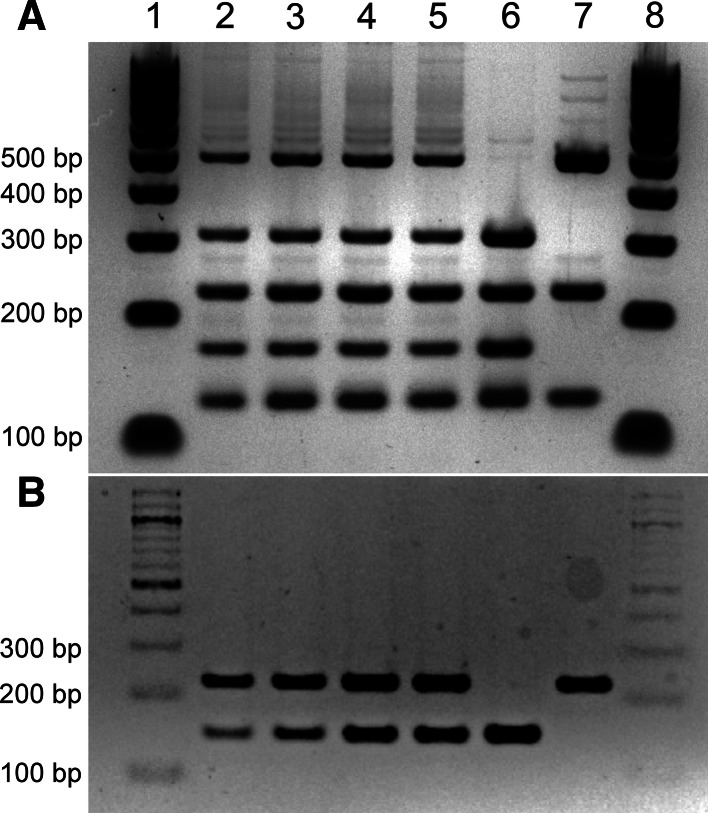
Confirmation of hybridization by **a** rDNA ITS PCR and RFLP, and **b** amplification of *FSY1* and *MEX67* genes. *Lanes 1 and 8*, 100 bp DNA ladder, *lane 2–5* hybrids H1–H4, *lane 6* *S. cerevisiae* A81062 parental strain, and *lane 7 S. eubayanus* C12902 parental strain

**Fig. 2:**
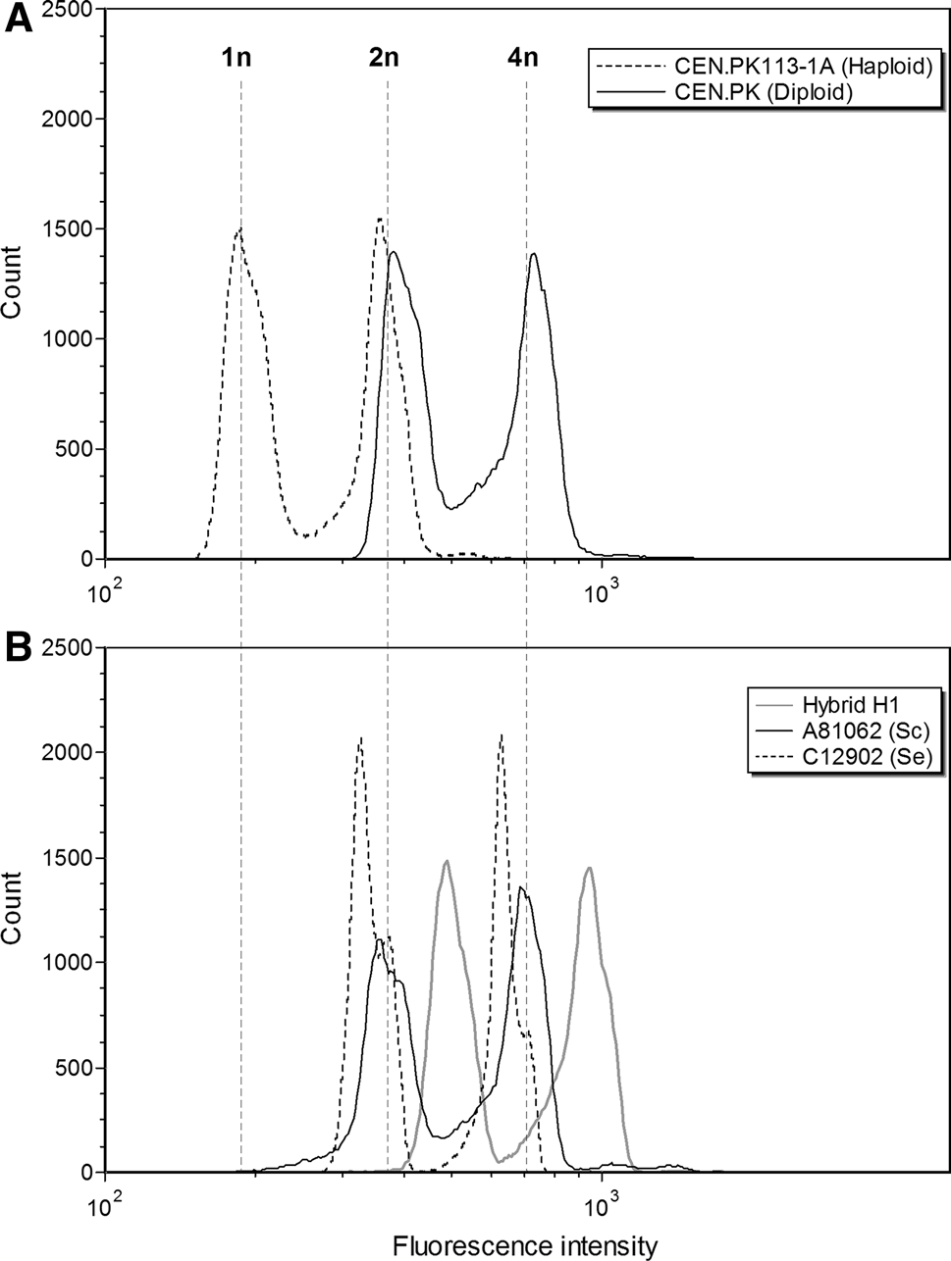
DNA content of the **a** *S. cerevisiae* haploid (CEN.PK113-1A) and diploid (CEN.PK) reference strains, and **b** hybrid strain H1 and parent strains by flow cytometry. The other hybrid strains H2–H4 showed identical profiles to that of hybrid H1. The *dashed vertical lines* represent the approximate fluorescence intensity corresponding to 1n, 2n and 4n DNA content

After amplification of the rDNA and digestion with *Hae*III, a 4-band pattern was obtained for the *S. cerevisiae* A81062 parental strain, a 3-band pattern was obtained for the *S. eubayanus* C12902, while 5-band patterns were obtained for the isolated hybrids (Fig. 1a), which confirms that both parental genomes were represented in these hybrids. This RFLP profile of the hybrids is different to that of industrial *S. pastorianus* hybrids which, as a result of post-hybridization DNA loss, exhibit either the 3-band or 4-band pattern depending on whether they belong to the Saaz or Frohberg group [14, 31]. Amplification of *FSY1* and *MEX67* genes by PCR (Fig. 1b), also revealed the presence of *S. eubayanus* and *S. cerevisiae* genes, respectively, in the genomes of the hybrid strains. Hybrid status was then finally confirmed with PFGE, which also suggested that the hybrid strains had inherited a complete set of chromosomes from both parent strains (shown in Supplementary material; Figure S1). Flow cytometry further revealed that all four hybrid strains were most likely triploid (Fig. 2). For clarity, only the fluorescence profile of hybrid strain H1 (of the hybrid strains) is shown in Fig. 2b, but the other hybrid strains H2–H4 produced identical profiles. This is in contrast to what would be expected from haploid spore-to-spore matings, which result in the formation of diploid cells. Here, hybridization was apparently a result of rare-mating between a haploid spore from one parent and a diploid cell, which may have undergone loss of heterozygosity and conversion to *a*/*a* or *α*/*α* mating type, from the other parent [15]. This also explains the relatively low hybridization frequency [1]. It is not known what portions of the parent genomes have been inherited in the hybrid strains, but it is likely that the four hybrid strains H1–H4 contained 2n DNA from the *S. cerevisiae* A81062 strain and 1n DNA from the *S. eubayanus* C12902 strain, because of the higher sporulation efficiency and spore viability of C12902. However, the data collected here are not enough to show this, and it is also possible that the hybrid strains contain proportionally more DNA from the *S. eubayanus* parent. The proportionality of DNA inheritance might be directly reflected in the functional properties of the hybrid strains, as seems to be the case for Saaz and Frohberg lager yeasts [13].

### Fermentation characteristics of hybrid strains

**Fig. 3:**
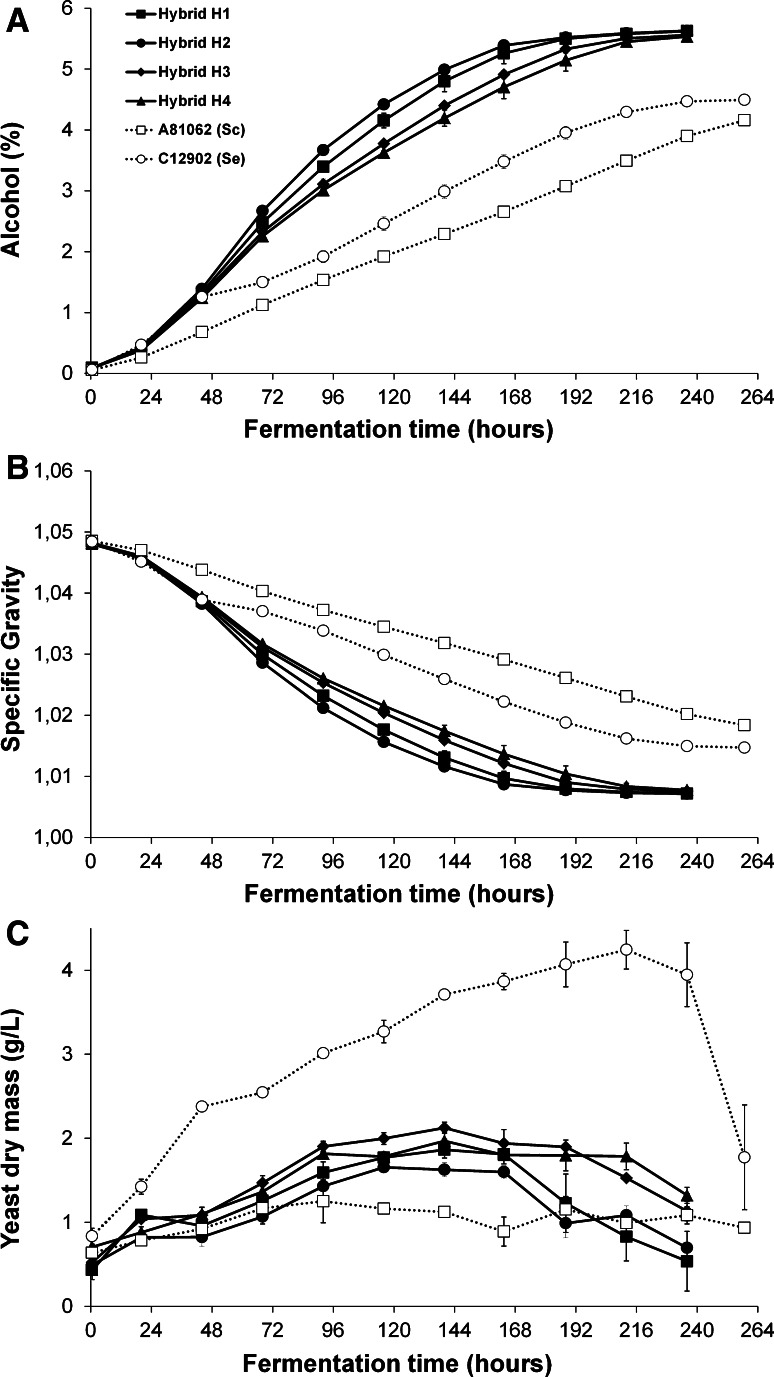
**a** The alcohol content (% ABV), **b** specific gravity and **c** suspended yeast dry mass (g/L) of the 12 °P wort fermented with the hybrid strains (*solid lines*) and parent strains (*dotted lines*). Values are means from two independent fermentations and *error bars* where visible represent the range

All four hybrid strains (H1–H4) successfully fermented the wort at 12 °C and displayed fermentation rates higher than both parent strains (Fig. 3). Fermentations with hybrid strains H1 and H2 were complete after 8 days. Not only did the hybrid strains ferment faster, but they also produced beers with higher alcohol content (5.6 vs 4.5 % ABV after 11 days of fermentation). The *S. cerevisiae* A81062 parent strain grew and fermented slowly at 12 °C and hence only reached an ABV of 4.2 % after 11 days. The successful growth and fermentation of the hybrid strains at 12 °C suggest that the cold tolerance of *S. eubayanus* has been transferred to the hybrid strains. The mechanisms that govern *S. eubayanus* cryotolerance are unknown, but it is most likely related to differences in the membrane composition [17] as well as product activity and expression of central metabolic genes [29], compared to other *Saccharomyces* yeast. Similar trends in fermentation profiles were observed during the ‘Generation 0’ fermentation performed at 20 °C, as the hybrid strains exhibited improved fermentation rates compared to both parent strains (data not shown). Furthermore, all four hybrid strains and the A81062 parent strain grew at 37 °C, but not 40 °C, on YPM plates, while *S. eubayanus* C12902 grew at neither temperature, suggesting the hybrid strains also inherited heat tolerance from the ale parent. The cause of the apparent heterotic phenotype of the hybrids could be differential functionality and expression of orthologous genes derived from the two parents (specifically linked to central carbon metabolism and sugar transport), as well as different temperature optima of the gene products [7, 14]. The small difference in fermentation performance between the hybrid strains could be a result of meiotic segregation during spore formation or differential inheritance of mitochondrial DNA, as mtDNA is inherited from only one parent [25, 35].

The sugar profiles of the original wort and the beers (Table 1) reveal that the higher attenuation reached by the hybrid strains compared to the *S. eubayanus* parent is a result of differential maltotriose utilization. The ability to ferment maltotriose seems to have been inherited from the *S. cerevisiae* parent, as the active sugar transport assays confirmed that all the hybrid strains and *S. cerevisiae* A81062 were able to transport this sugar across the cell membrane, while *S. eubayanus* C12902 showed negligible uptake (Table 2) as shown previously by Gibson et al. [13]. Maltotriose uptake is common in lager yeast strains; however, this ability seems to be limited to strains belonging to the Frohberg group [13]. Maltose and maltotriose use during fermentation are dependent on the activity of a range of transmembrane transporters [46]. In industrial ale strains maltotriose is essentially carried into the cell by *AGT1* transporters, while it has been found in lager yeast strains that *S. cerevisiae*-derived *AGT1* genes remain non-functional, and maltotriose transport is rather thought to be carried out by Mtt1 (Mty1) and a diverged form of *AGT1*, which have presumably both been inherited from the *S. eubayanus* parent [10, 45, 46, 48]. This contradicts the fact that negligible transport of maltotriose into the *S. eubayanus* type strain was observed here and in previous studies [13], but the maltotriose use of other *S. eubayanus* strains, e.g. from different geographical origins, currently remains unexplored. The utilization of maltotriose is of importance during brewery fermentations because it is the second most abundant sugar in wort, its utilization results in a higher alcohol yield and residual maltotriose can affect flavour [51]. The fermentation profiles (Fig. 3a, b) reveal that the hybrid strains and *S. eubayanus* exhibited a similar fermentation rate in the beginning of fermentation, when monosaccharides are predominately taken up and utilized by the yeast [3]. After the first 48 h, the fermentation rates of the hybrid strains increase relative to *S. eubayanus*, presumably because of more efficient maltose and maltotriose utilization in the former. The hybrid strains and *S. eubayanus* exhibited fermentation rates greater than *S. cerevisiae* during essentially the entire fermentation because the temperature is clearly sub-optimal for *S. cerevisiae*. This is also evident from the fact that there was residual maltose in the beer fermented with *S. cerevisiae* (Table 1). Here, a relatively low-gravity wort (12 ° Plato) was used, but industry is showing interest towards higher gravity worts [37]. While the fermentation performance of the hybrid strains in high-gravity wort was not tested in this study, it may be possible that they possess increased stress tolerance as a result of their polyploidy [52].

**Table 1:** Sugars (g/L) in the original wort and beers fermented with the hybrid and parent strains

Yeast strain/sample	Maltose	Maltotriose
Original wort	59.1 (±0.37)	18.8 (±0.21)
Hybrid H1	1.6 (±0.04)	3.6 (±0.07)
Hybrid H2	1.5 (±0.03)	3.4 (±0.05)
Hybrid H3	1.6 (±0.02)	3.9 (±0.13)
Hybrid H4	1.6 (±0.20)	5.3 (±0.24)
A81062	25.1 (±0.69)	5.5 (±0.09)
C12902	1.8 (±0.15)	18.5 (±0.58)

Values are means from two independent fermentations (standard deviation in parenthesis)

**Table 2:** The flocculation ability and maltose and maltotriose uptake at 20 °C by the hybrid and parent strains

Yeast strain	Flocculation ability (%)	Maltose uptake µmol min^−1 ^g^−1^ DY (5 mM maltose)	Maltotriose uptake µmol min^−1 ^g^−1^ DY (5 mM maltotriose)
Hybrid H1	85 (±2.1)	12.6 (±1.7)	4.1 (±0.3)
Hybrid H2	87 (±1.3)	12.3 (±3.0)	4.1 (±0.2)
Hybrid H3	88 (±1.6)	15.8 (±0.9)	5.4 (±1.4)
Hybrid H4	82 (±4.5)	16.1 (±0.7)	5.4 (±1.3)
A81062	71 (±4.2)	10.1 (±0.5)	4.2 (±0.2)
C12902	15 (±0.8)	13.2 (±3.1)	0.1 (±0.0)

An uptake activity  ≤0.5 µmol min^−1 ^g^−1^ DY is considered negligible. Values are means of three independent assays (standard deviation in parenthesis)

**Fig. 4:**
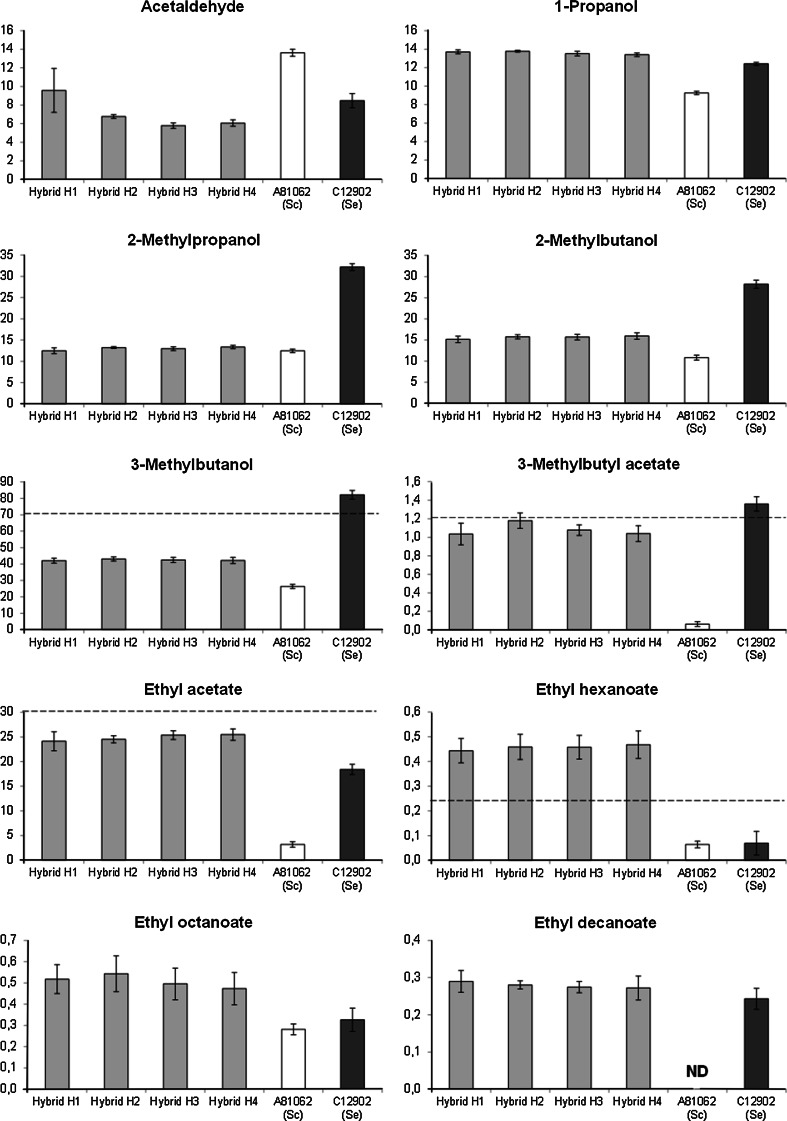
The concentrations of aroma compounds in the beers fermented with the hybrid and parent strains (mg/L). Where visible, the *dashed line* represents the typical flavour threshold [26]. Values are means from two independent fermentations and *error bars* where visible represent the range

The concentrations of aroma compounds in the beers (Fig. 4) reveal that all four hybrid strains produced beers with similar aroma profiles. Compared to the hybrid strains, *S. eubayanus* C12902 produced more of the higher alcohols related to branched-chain amino acid synthesis (almost twofold concentrations of 2-methylpropanol, 3-methylbutanol and 2-methylbutanol). These give the beer alcoholic and solvent-like aromas, which are generally considered unpleasant [32]. Despite these twofold differences in 3-methylbutanol concentrations, the concentrations of 3-methylbutyl acetate were quite similar for the hybrid strains and *S. eubayanus* (around the flavour threshold of 1.2 mg/L [26]). 3-Methylbutyl acetate gives beer a banana- and pear-like aroma, which is considered desirable in several beer styles [32]. The hybrid strains produced more ethyl esters than both parent strains, and the concentrations of ethyl hexanoate were above the flavour threshold of 0.2 mg/L [26]. Ethyl esters give beer a fruity and apple-like aroma. The cause of these differences in ester formation of the hybrid strains compared to both parent strains is not known, but could be due to both genetic differences (e.g. increased expression and different functionality of orthologous genes [5, 37, 44]) and an indirect result of the fermentations, e.g. from the formation of more alcohol and fatty acid precursors, differences in wort pH or differences in yeast growth [18, 49]. The formation of acetate esters is dependent mainly on the expression and enzyme activities of the transferase-encoding *ATF1* and *ATF2* genes [44], while Saerens et al. [36, 37] found that the enzymes encoded by *EHT1* and *EEB1* genes are mainly responsible for ethyl ester synthesis. Overall, a low amount of esters was observed in the beer fermented with *S. cerevisiae* A81062. This was most likely a result of the low and sub-optimal fermentation temperature, resulting in less expression and enzyme activity of genes involved with ester synthesis, e.g. *ATF1, ATF2, EEB1* and *EHT1*. It has been shown that the expression of these genes, encoding transferase enzymes involved in acetate and ethyl ester synthesis, is increased at increasing temperatures [37, 44]. The results suggest that it could be possible to increase aromatic diversity of lager yeast strains through interspecific hybridization and produce lager yeast strains with desired aroma production through the selection of appropriate parent strains. However, more work on the subject, especially in regard to relationships between orthologous gene expression and aroma production, must still be done. All beers also had a distinct clove-like aroma, caused by the presence of 4-vinylguaiacol [8], suggesting that the hybrid strains had inherited the *PAD1* gene, coding for the phenylacrylic acid decarboxylase enzyme responsible for the conversion of ferulic acid to 4-vinylguaiacol, from either of the parents, which were both *PAD1*+ (our own unpublished data).

The flocculation assay revealed strong flocculation for both the *S. cerevisiae* A81062 parent strain and all four hybrid strains H1–H4, while *S. eubayanus* C12902 displayed poor flocculation (Table 2). These properties were also evident during fermentation, as more suspended biomass was observed in the fermentations with *S. eubayanus* than with the hybrids (Fig. 3c). The flocculation ability of yeast is defined mainly by which *FLO* genes are functional in various strains [47], and here the hybrid strains have clearly inherited flocculation ability from the *S. cerevisiae* parent. Surprisingly, the flocculation of the hybrid strains was stronger than both the parent strains, thus again revealing phenotype amplification (Table 2). Flocculation of yeast towards the end of fermentation allows for a cheap and effective way of removing yeast from the beer, and the use of strongly flocculating yeast strains is especially popular among smaller breweries.

In conclusion, the use of mass mating to generate de novo lager yeast hybrids through interspecific hybridization between a *S. cerevisiae* ale strain and *S. eubayanus* was successful. The resulting hybrid strains not only inherited beneficial properties from both parent strains (cryotolerance, maltotriose utilization and strong flocculation), but also showed apparent hybrid vigour compared to the parent strains by fermenting faster and achieving a more complete utilization of fermentable sugars. It must be mentioned that here, the performance of the hybrid strains was compared relative to the original parental strains and not the actual haploid spore product that mated to form the triploid hybrids. The possible heterozygosity of the parent strains suggests that spore products may be quite different genetically from any other spore product and thus also have quite different fermentation behaviour than either the parent or other sister spores [2, 41]. How natural lager yeast hybrids were first created is still not known. The clearly improved fermentation performance of the hybrid strains shown here would support the hypothesis that *S. eubayanus* may have initially been present as a contaminant in *S. cerevisiae* ale fermentations. At lower temperatures the hybrid state, which confers phenotypic benefits from both parents, would have been selected for and hybrids would have quickly dominated the brewing process [14, 42]. The genomes of newly created yeast hybrids are also known to be unstable, and this may allow for evolutionary adaptation of hybrids to different environments [20]. These results suggest that interspecific hybridization is suitable for production of novel non-GM lager yeast strains with unique properties (e.g. flavour production or elevated stress tolerance) and generation of novel beer styles. Further investigation of the properties of de novo hybrids is required, especially in regard to the proportionality of DNA inheritance, the origin of mitochondrial DNA and aroma production and may help to elucidate the evolutionary history of industrial lager yeast strains. As a final remark, we want to mention the recent work of Marit Hebly and co-workers from Delft University of Technology entitled “*Saccharomyces cerevisiae* x *Saccharomyces eubayanus* interspecific hybrid, the best of both worlds and beyond” that further reinforces our observations and ideally complements our study. This work will be published shortly in FEMS Yeast Research.

## Electronic supplementary material

Below is the link to the electronic supplementary material.

Supplementary material 1 (PDF 1123 kb)
