# Supplementary material for: New lager yeast strains generated by interspecific hybridization
Source: J Ind Microbiol Biotechnol. 2015 Feb 15;42(5):769–78. doi: 10.1007/s10295-015-1597-6 (PMC4412690; doi:10.1007/s10295-015-1597-6)
Supplement: Supplementary file 1 — Supplementary material 1 (PDF 1123 kb) [file 10295_2015_1597_MOESM1_ESM.pdf]

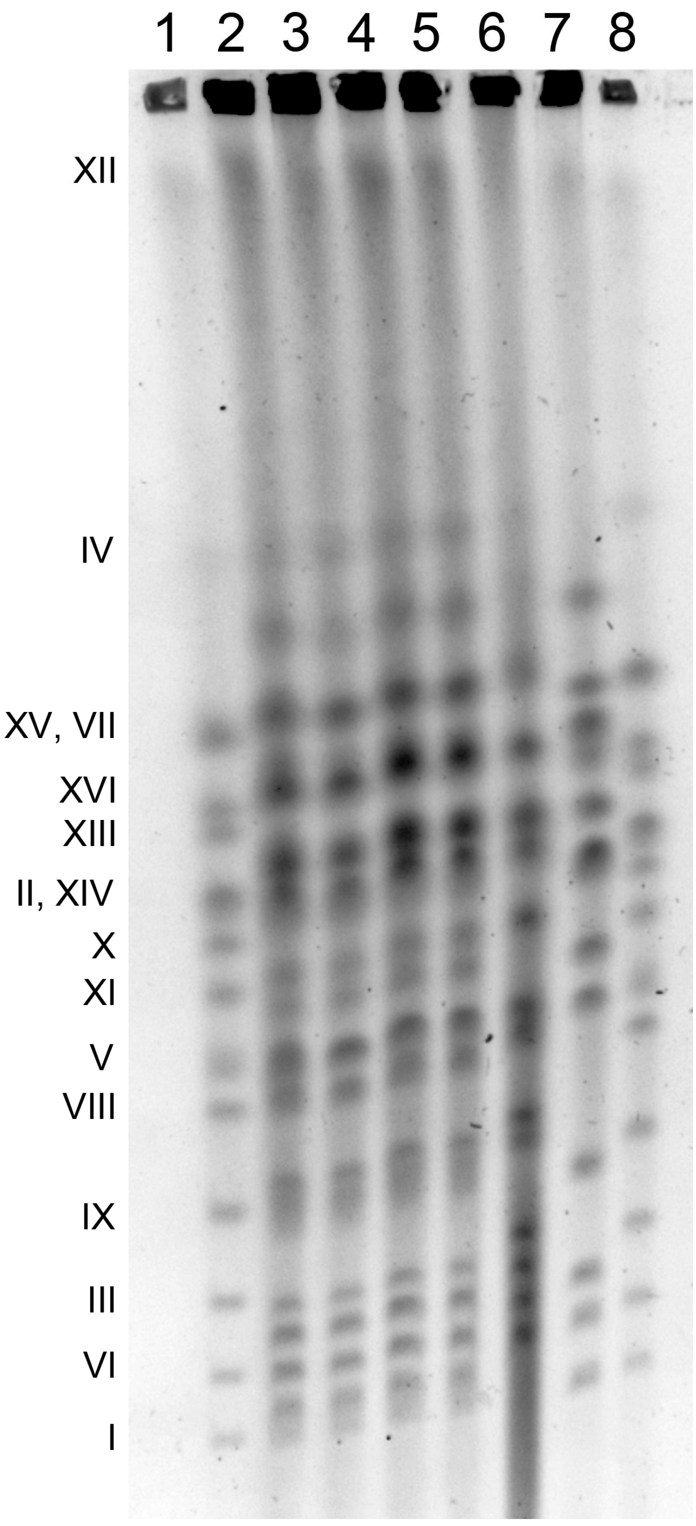

Figure S1. PFGE separation of chromosomes from hybrid strains H1-H4 and parent strains. Lanes 1 & 8 chromosome marker strain YNN295, lane 2-5 hybrids H1-H4, lane 6 *S. cerevisiae* A81062 parental strain, and lane 7 *S. eubayanus* C12902 parental strain. Chromosomes are identified on the left: chromosomes VII and XV are not resolved; chromosome II travels immediately above chromosome XIV.
